# Artemisitene Ameliorates Diabetic Wounds by Inhibiting Ferroptosis Through Activation of the Nrf2/GPX4 Pathway

**DOI:** 10.1002/fsn3.70952

**Published:** 2025-09-17

**Authors:** Xu Honghao, Bu Yitian, Zhao Yuan, Long Zhengyang, Zhou Feiya, Cai Leyi, Gao Weiyang, Wang Anyuan, Wu Hongqiang

**Affiliations:** ^1^ Department of Orthopaedics The Second Affiliated Hospital and Yuying Children's Hospital of Wenzhou Medical University Wenzhou China; ^2^ Key Laboratory of Orthopaedics of Zhejiang Province Wenzhou China; ^3^ Department of Ultrasonic Diagnosis The Second Affiliated Hospital and Yuying Children's Hospital of Wenzhou Medical University Wenzhou China; ^4^ Wenzhou Medical University Wenzhou China

**Keywords:** artemisitene, diabetic wound healing, ferroptosis, Nrf2/GPX4 pathway, oxidative stress

## Abstract

Diabetic wounds are hard to heal due to ferroptosis, a specific type of cell death triggered by high blood sugar in human umbilical vein endothelial cells (HUVECs) in the lining of blood vessels. Ferroptosis occurs as a result of excessive iron accumulation and lipid peroxidation. The resulting impairment in endothelial function and tissue scaffolding creates a persistent barrier against effective wound healing. Our study found that artemisitene (ATT), a bioactive compound derived from the herb *Artemisia annua* , speeded up diabetic wound healing by blocking ferroptosis through the Nrf2 (NFE2 like bZIP transcription factor 2, also known as NRF2: Nuclear factor erythroid 2‐related factor 2)/GPX4 (Glutathione peroxidase 4) pathway. In studies with HG (high glucose)‐damaged HUVECs, treatment with ATT (20 μM) effectively counteracted harmful iron buildup and lipid peroxidation, while also restoring mitochondrial health and reducing the levels of damaging reactive oxygen species (ROS). Computational modeling confirmed that ATT binds tightly to both Nrf2 and GPX4 molecules. Notably, when Nrf2 was blocked, ATT completely lost its protective effect, indicating that Nrf2 is essential for its action. In diabetic rats, applying ATT directly to wounds (20 mg/kg/day) significantly accelerated the rate of wound closure. This treatment worked by triggering two key regenerative processes: stronger new blood vessel growth and better‐organized collagen structure. In practical terms, ATT prevents diabetic wound complications through three connected mechanisms: it uses GPX4 to counteract lipid peroxides, leverages Nrf2 to restore healthy antioxidant balance, and regenerates endothelial cells to drive new blood vessel formation. As the first drug of its kind to target ferroptosis this way, ATT offers a promising multitarget approach for diabetic wounds, tackling the combined problems of oxidative damage, mitochondrial failure, and poor tissue regeneration.

## Graphical abstract

Artemisitene (ATT), an artemisinin derivative, alleviates high glucose‐induced ferroptosis in endothelial cells by activating the Nrf2/GPX4 pathway, thereby reducing lipid peroxidation and oxidative stress. In diabetic rats, ATT accelerates wound healing through enhanced angiogenesis and GPX4‐mediated ferroptosis suppression. These findings reveal ATT's dual antioxidant/anti‐inflammatory role in diabetic complications, positioning it as a promising therapeutic candidate.

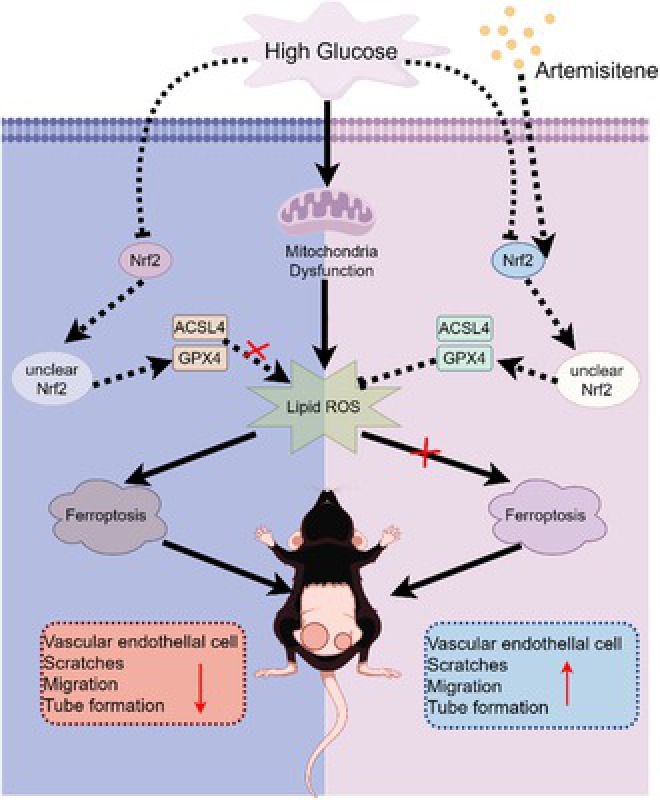

## 1 Introduction

Diabetic wounds are one of the most common and serious complications of diabetes mellitus, and their formation is closely associated with various pathophysiological mechanisms (Zheng et al. 2025; Humphries 2025; Lou et al. 2025). Typically, diabetic wounds manifest as skin tissue damage, infection, and ulceration (Joshi et al. 2025). The etiology of diabetic wounds is complex, involving factors such as poor glycemic control, neuropathy, vascular dysfunction, and impaired immune function (McGloin et al. 2021). Vascular pathology is the core pathological basis of the refractory nature of diabetic wounds, including macrovascular complications (e.g., atherosclerosis) and microvascular complications (e.g., basement membrane thickening and endothelial cell injury) (Yan et al. 2003; Wirostko et al. 2008). These pathological changes lead to circulatory disturbances, further exacerbating tissue ischemia and hypoxia, thereby delaying wound healing (Huang et al. 2023). The treatment of diabetic wounds faces numerous challenges, including difficulties in ulcer healing, infection control, and complex pain management (Li, Lai, et al. 2025). Treatment options are limited, with current approaches primarily including pharmacological therapy, physical therapy, and surgical interventions (She et al. 2025). In pharmacological therapy, antibiotics are used for infection prevention and treatment, anticoagulants improve blood circulation, and growth factor‐based drugs promote cell proliferation and wound healing (Perussolo et al. 2025). Physical therapies, such as negative pressure wound therapy and phototherapy, as well as surgical procedures like debridement and skin flap transplantation, are also widely used in clinical practice (Capobianco et al. 2010). However, due to the complexity and individual variability of diabetic wounds, treatment outcomes often vary considerably, and more effective therapeutic strategies need to be further explored.

Persistent inflammation, impaired angiogenesis, and excessive oxidative stress within the diabetic microenvironment impede wound healing (Mao et al. 2025). This pathology arises from severe redox imbalance: uncontrolled generation of ROS through mitochondrial dysfunction and enzymatic sources (e.g., NADPH oxidase), concomitant with critical depletion of glutathione reserves and inactivation of core antioxidant defenses, including superoxide dismutase and catalase (Xiong et al. 2023; Accipe et al. 2023). Recent evidence indicates that the impaired healing of diabetic wounds is closely associated with ferroptosis (Zhang et al. 2024). Under hyperglycemic conditions, dysregulated iron metabolism and excessive lipid peroxidation lead to cellular membrane damage and exacerbated inflammatory responses, further preventing wound repair (Feng et al. 2022). The role of the Nrf2/GPX4 signaling pathway in ferroptosis and impaired diabetic wound healing has garnered significant attention in recent years (Shi et al. 2023). Ferroptosis, an iron‐dependent, lipid peroxidation‐driven form of cell death, is characterized by reduced GPX4 activity and the accumulation of lipid peroxides (Lin et al. 2025). Nrf2, a key regulator of cellular responses, inhibits ferroptosis by regulating the expression of antioxidant genes, including *GPX4* (Schmidt et al. 2025). In diabetic wounds, hyperglycemia and metabolic disturbances exacerbate oxidative stress, impairing the function of the Nrf2/GPX4 pathway and thereby promoting ferroptosis, which delays wound healing (Gao et al. 2022). Therefore, the development of therapeutic strategies combining antioxidant treatments with ferroptosis inhibitors is considered a promising approach for improving diabetic wound management.

Artemisitene (ATT), a bioactive compound derived from *Artemisia annua* distinct from artemisinin, has documented redox‐modulating and anti‐inflammatory properties, alongside its known antimalarial/anticancer activities (Gao et al. 2024; Ooko et al. 2015; Efferth et al. 2011). Previous studies by Bai et al. showed that ATT significantly alleviates sepsis‐induced liver injury by inhibiting ferroptosis. Liu et al. found that ATT enhances the cellular antioxidant defense by stabilizing Nrf2, thereby protecting against bleomycin‐induced lung injury (Chen et al. 2016). Its therapeutic potential specifically targeting diabetic wounds, particularly concerning ferroptosis modulation, remains unexplored, despite its natural multi‐targeting ability.

This study shows that ATT significantly enhances diabetic wound healing in streptozotocin (STZ)‐induced diabetic mice and rescues hyperglycemia‐damaged human endothelial cells. Mechanistically, ATT activates the Nrf2 pathway, upregulating GPX4 expression. This activity simultaneously reduces oxidative stress, ferroptotic cell death, and inflammation. Thus, ATT, acting through the Nrf2/GPX4 signaling pathway, represents a novel multifunctional therapeutic agent capable of disrupting core pathological networks in diabetic wounds, establishing targeted modulation of redox‐ferroptosis crosstalk as a strategic therapeutic approach for diabetic wound healing.

## 2 Materials and Methods

### 2.1 Reagents and Antibodies

Artemisitene/ATT (HY‐122550; 99.43% purity), the ferroptosis inducer erastin, and the selective ferroptosis inhibitor ferrostatin‐1 (Fer‐1) were purchased from MedChemExpress (Monmouth Junction, NJ, USA). Primary antibodies used in this study included: anti‐ACSL4 (ab155282; 1:1000), anti‐GPX4 (ab252833; 1:1000), and anti‐GAPDH (9485; 1:3000) obtained from Abcam (Cambridge, UK); anti‐Nrf2 (16396‐1‐AP; 1:1000), anti‐VEGFA (19003‐1‐AP; 1:1000), and anti‐Lamin B1 (12987‐1‐AP; 1:2000) obtained from Proteintech Group Inc. (Rosemont, IL, USA). The fluorescent dye 4′,6‐diamidino‐2‐phenylindole (DAPI) was purchased from Beyotime (Shanghai, China). Additional reagents used in this study included: the 5‐ethynyl‐2′‐deoxyuridine (EdU) assay kit (Elabscience, Wuhan, China), the fluorescent probe C11‐BODIPY (Invitrogen, Carlsbad, CA, USA), the fluorescent probe FerroOrange (Dojindo, Shanghai, China), Dulbecco's modified Eagle's medium (DMEM), phosphate‐buffered saline (PBS), fetal bovine serum (FBS), and 0.25% trypsin solution (Gibco/Thermo Fisher Scientific Inc., Waltham, MA, USA). All other chemicals were procured from MilliporeSigma (Burlington, MA, USA).

### 2.2 Cell Culture Protocol

Human umbilical vein endothelial cells (HUVECs) were obtained from the American Type Culture Collection (ATCC, Manassas, VA) and maintained in DMEM supplemented with 10% heat‐inactivated FBS, 1% penicillin/streptomycin (v/v) under standard cell culture conditions (37°C, 5% CO_2_) in a humidified incubator. Following expansion in T‐75 flasks (Falcon) at a density of 2500–3000 cells/cm^2^, cell culture medium was replenished every 48 h. Cellular confluence (> 90%) was routinely achieved within 6–7 days, as verified by phase‐contrast microscopy using a Nikon Eclipse TS100 Inverted Microscope (Nikon Corporation, Tokyo, Japan), at which point cells were detached using 0.25% trypsin–EDTA and passaged for use in subsequent experiments.

### 2.3 Cell Culture Protocols

HUVECs were cultured in DMEM supplemented with 10% heat‐inactivated FBS and 1% penicillin/streptomycin (v/v) under standard cell culture conditions (37°C, 5% CO_2_) in a humidified incubator. Cells were seeded in Corning Falcon T‐75 flasks (Corning Life Sciences, Corning, NY, USA) at a density of 2500–3000 cells/cm^2^, replenishing complete medium every 48 h. Phase‐contrast microscopy using a Nikon Eclipse TS100 Inverted Microscope (Nikon Corporation) confirmed that > 90% cellular confluence was routinely attained within 6‐7 days. Confluent monolayers were subsequently detached using Gibco 0.25% trypsin–EDTA (Thermo Fisher Scientific Inc.) and subcultured for use in subsequent experiments.

### 2.4 Dihydroethidium (DHE) Staining

Cells were incubated with 5 μM DHE (MilliporeSigma) in serum‐free medium for 30 min at 37°C in the dark. After washing with PBS, live‐cell imaging was immediately performed using a Zeiss LSM 900 laser scanning confocal microscope (Carl Zeiss Microscopy GmbH, Oberkochen, Germany) equipped with 488 nm excitation/590 nm emission filters. Fluorescence intensity was quantified using the ImageJ software (National Institute of Health (NIH), Bethesda, MD, USA).

### 2.5 Transmission Electron Microscopy (TEM)

Cellular ultrastructure was examined by TEM. After overnight fixation at 4°C, cells were postfixed with 1% osmium tetroxide (OsO_4_) for 30 min. Following dehydration through an ethanol series and embedding, ultrathin sections were prepared and stained with uranyl acetate and lead citrate. Sections were examined using a Hitachi transmission electron microscope (Hitachi Corporation, Tokyo, Japan).

### 2.6 EdU Proliferation Assay

HUVECs were pulsed with 10 μM EdU (2 h, 37°C), fixed/permeabilized (4% PFA, 0.5% Triton X‐100), and labeled via Click‐iT reaction (Alexa Fluor 488, 30 min in the dark). After DAPI staining, laser scanning confocal microscopy images were acquired using a Zeiss LSM 900 confocal scanning microscope, and EdU^+^ cells were counted in random fields.

### 2.7 Western Blot Analysis

After washing with ice‐cold PBS, HUVECs were lysed in radioimmunoprecipitation assay (RIPA) buffer (Beyotime) containing protease/phosphatase inhibitors (MilliporeSigma), and lysate supernatants were collected by centrifugation at 12,000 × g. Then, aliquots containing 20 μg of protein were separated by sodium dodecyl sulfate‐polyacrylamide gel electrophoresis (SDS‐PAGE) on a 10% acrylamide gel, and the separated proteins were transferred to polyvinylidene fluoride (PVDF) membranes. After blocking with 5% skim milk, membranes were incubated with the corresponding primary antibodies at 4°C overnight. Subsequently, after washing with PBS, the membranes were incubated with the appropriate horseradish peroxidase (HRP)‐conjugated secondary antibodies (1:5000) at room temperature for 1 h. Immunoreactive protein bands were visualized using enhanced chemiluminescence (ECL) reagent for labeling and the Bio‐Rad ChemiDoc XRS+ system (Bio‐Rad Laboratories Inc., Hercules, CA, USA) for chemiluminescence imaging. Immunoreactive protein bands on the images were quantified using the Image Lab 6.0 software (Bio‐Rad Laboratories Inc.) and normalized to the GAPDH loading control.

### 2.8 Cell Viability Assay

HUVECs viability was assessed using a cell counting kit‐8 (CCK‐8) assay kit (Elabscience, Wuhan, China). After the various treatments, cells were incubated with CCK‐8 working solution (10% reagent) for 1‐2 days, followed by 1 h at 37°C. Absorbance was measured at 450 nm using a Multiskan MK3 microplate reader (Thermo Fisher Scientific Inc.).

### 2.9 C11‐BODIPY and FerroOrange Staining

Lipid peroxidation levels were assessed using C11‐BODIPY (Invitrogen). After treatment, HUVECs were incubated with 1 μM C11‐BODIPY working solution for 30 min. Intracellular Fe^2+^ was detected using a FerroOrange probe (Dojindo). After washing with PBS, HUVECs were incubated with 1 μM FerroOrange working solution for 30 min. Stained cells were imaged using a Carl Zeiss LSM 900 confocal scanning microscope.

### 2.10 MitoSOX Staining for Mitochondrial Superoxide Radicals

HUVECs were incubated with 5 μM MitoSOX Red (Thermo Fisher Scientific Inc.) in serum‐free medium for 30 min at 37°C in the dark. After washing with PBS, cells were immediately imaged using Zeiss LSM 900. Red fluorescence intensity correlated with mitochondrial superoxide production.

### 2.11 JC‐1 Staining for Mitochondrial Membrane Potential (ΔΨm)

HUVECs were incubated with 2 μM JC‐1 (Thermo Fisher Scientific Inc.) in serum‐free medium for 30 min at 37°C under light‐protected conditions. After PBS washing, cells were immediately imaged using a Zeiss LSM 900 confocal scanning microscope. Mitochondria with intact mitochondrial membrane potential (ΔΨm) showed red fluorescence (J‐aggregates), while depolarized mitochondria showed green fluorescence (monomers).

### 2.12 Tube Formation Assay

HUVEC tube formation was assessed on matrix‐coated chamber slides, which were prepared by adding the ECMatrix gel solution to the u‐slide plate and allowing it to solidify by incubating it at 37°C for 1 h. Pre‐treated HUVECs were harvested using trypsin/EDTA, centrifuged at 200 × g for 5 min, and seeded on the solidified matrix. Then, the plate was incubated at 37°C for 6 h to allow tube formation. Resulting tube networks were visualized by phase contrast microscopy and quantified by measuring the percentage of area covered by tubes in each well.

### 2.13 Cell Migration Assay

Migratory capacity of HUVECs was assessed using Corning Costar plates with Transwell chambers with 8‐μm pore permeable membrane inserts (Corning Life Sciences). Isovitexin‐treated cells were serum‐starved in DMEM for 4 h prior to seeding (1 × 10^4^ cells/well in 200 μL serum‐free medium) into the upper chambers. Lower chambers contained 700 μL DMEM with 1% FBS as chemoattractant. After incubation for 12 h under standard cell culture conditions (37°C, 5% CO_2_), membrane inserts were fixed with 4% paraformaldehyde (PFA), washed with PBS, and stained with 0.1% crystal violet. Non‐migratory cells were mechanically removed from upper surfaces using cellulose swabs.

### 2.14 Wound Healing Assay

Confluent HUVEC monolayers in 6‐well plates were scratched with sterile pipette tips and treated in serum‐free DMEM under four experimental conditions: ATT plus HG, HG alone, HG plus Fer‐1, and untreated controls. After incubation (37°C, 5% CO_2_) for 12 h, wounds were imaged at 0 and 21 h by bright‐field microscopy using an Olympus CKX53 light microscope (Olympus Corporation, Tokyo, Japan). Migration distance was determined by measuring gap widths at three fixed positions per wound using the ImageJ software (NIH).

### 2.15 Immunofluorescence Staining

Cells on coverslips were fixed in 4% PFA (15 min, room temperature), permeabilized with 0.2% Triton X‐100 (10 min), then blocked in 5% bovine serum albumin (BSA) for 1 h. The appropriate primary antibodies were added onto the coverslips, which were then incubated overnight at 4°C, followed by incubation with Alexa Fluor‐conjugated secondary antibodies (1 h, room temperature, in the dark). After staining nuclei with DAPI for 5 min, images were acquired using a Zeiss LSM 900 confocal scanning microscope.

### 2.16 Molecular Docking Analysis

The ATT structure was sketched in ChemBioDraw and energy‐minimized using ChemBio3D. The Nrf2 and GPX4 protein structures were retrieved from the Universal Protein Resource (UniProt) database. Docking conformations were generated in PyMOL using default parameters to achieve minimal energy states. Using AutoDock Tools 1.5.6, protein and ligand files were converted to PDBQT format. Active site identification and search space definition were performed in AutoDock Tools, followed by ligand‐receptor docking execution in AutoDock Vina 1.2.1. Results were visualized with Discovery Studio.

### 2.17 Small Interference RNA (siRNA) Transfection

The *Nrf2*‐targeting siRNA (Invitrogen) was transfected into HUVECs at 30%–50% confluence. After incubation for 12 h (cell viability > 95%), fresh medium was replenished. Cells were cultured for 72 h prior to assays. Transfection efficiency was confirmed by Western blot analysis.

### 2.18 Ethical Grouping and Therapeutic Regimens in Experimental Animals

This study was approved by the Wenzhou Medical University Institutional Animal Care and Use Committee in strict compliance with AAALAC International accreditation standards and ARRIVE guidelines. Thirty 8‐week‐old specific pathogen‐free (SPF) C57BL/6 male mice were randomly assigned by stratified randomization into five experimental groups: Control, DM (diabetic microenvironment), DM+L‐ATT (10 mg/kg), DM+H‐ATT (20 mg/kg), and DM+Fer‐1 (5 mg/kg). Surgical anesthesia was induced by intraperitoneal injection of pentobarbital sodium (100 mg/kg) with corneal reflex monitoring. Bilateral 10‐mm diameter full‐thickness excisional wounds were created along the dorsal midline. Topical therapeutic intervention at wound margins was initiated 24 h postoperatively and administered twice daily until completion of the study. Wound photographs were captured on days 0, 3, 7, and 14, followed by appropriate treatment of the wound areas. The wound areas were measured using Image J software, and statistical analysis was subsequently performed.

### 2.19 Laser Doppler Perfusion Imaging

Wound blood flow was evaluated in anesthetized mice (2% pentobarbital sodium, i.p.) by laser Doppler imaging using a laser Doppler blood flow meter (Moor Instruments Ltd., Axminster, Devon, UK) at postoperative days 3, 7, and 14. Following dorsal hair removal, perfusion images of wound areas were captured. Quantitative analysis measured blood flow in perfusion units (PU) across three predefined regions per wound site.

### 2.20 Histopathological Evaluation

Skin specimens were fixed in 4% PFA for 24 h, paraffin embedded, and serial sections (4 μm thickness) were cut using a Leica RM2235 rotary microtome (Leica Biosystems, Nussloch, Germany). For comprehensive tissue architecture assessment, sections were stained with hematoxylin & eosin (H&E) (Beyotime) following standard protocols. Collagen deposition was quantitatively analyzed using a Masson's trichrome staining kit (Beyotime) according to the manufacturer's instructions.

### 2.21 Immunohistochemistry (IHC) Analysis

Tissue sections underwent antigen retrieval in citrate buffer (95°C, 20 min). After endogenous peroxidase blocking (3% H_2_O_2_, 10 min), sections were blocked with 5% BSA (30 min) and incubated with the appropriate primary antibody overnight (4°C). Subsequently, sections were incubated (1 h, RT) with the HRP‐conjugated secondary antibody, followed by diaminobenzidine staining (5 min). Counterstaining with hematoxylin preceded dehydration and mounting. Images were acquired by bright‐field microscopy using a Carl Zeiss Axio Imager (Carl Zeiss Microscopy GmbH).

### 2.22 Immunofluorescence Staining

Tissue sections underwent heat‐mediated antigen retrieval in citrate buffer (95°C, 20 min). After blocking with 10% serum (30 min, room temperature), sections were incubated overnight at 4°C with primary antibodies against α‐SMA and Nrf2, followed by species‐matched Alexa Fluor secondary antibodies (1 h, in the dark). After nuclear staining with DAPI, images were acquired using a Zeiss LSM 900 confocal scanning microscope. Fluorescence intensity was quantified in three random fields per section using threshold‐based segmentation.

## 3 Results

### 3.1 HG Microenvironment Induces Ferroptosis in HUVECs

**FIGURE 1:**
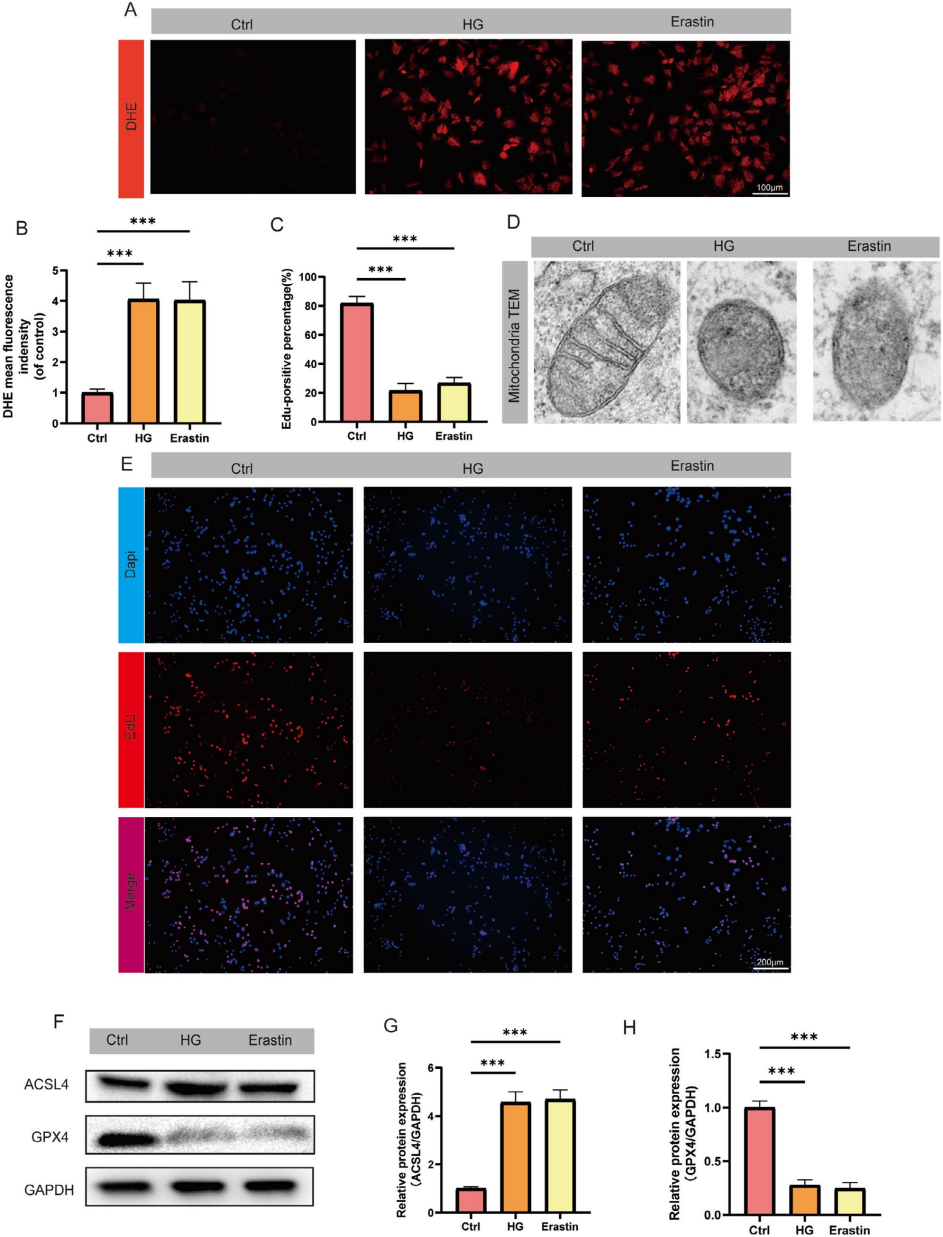
High glucose (HG) induces ferroptosis in HUVECs. (A) Representative DHE fluorescence staining images showing intracellular ROS levels in HUVECs treated with HG (25.5 mM) or erastin (5 μM) for 24 h. (B) Quantification of DHE fluorescence signal intensity. (C) Quantification of EdU+ cells from panel E. (D) TEM ultrastructural analysis of mitochondria in HG/erastin‐treated HUVECs. (E) EdU proliferation assay showing HG/erastin‐induced DNA synthesis inhibition. (F–H) Western blot analysis of GPX4 and ACSL4 expression. (***p < 0.001 vs. Ctrl; *n* = 5). Data expressed as the mean ± SD.

To mimic the diabetic microenvironment in vitro, HUVECs were cultured under HG and normal glucose (NG) conditions as control, following established protocols (Su et al. 2023). Microscopic analysis revealed that prolonged exposure to HG or the ferroptosis inducer erastin significantly increased the DHE fluorescence intensity, which is indicative of elevated intracellular ROS levels, accompanied by a marked increase in DHE‐positive cell populations (Figure 1A,B). TEM analysis further revealed characteristic ultrastructural changes in HG‐ and erastin‐treated HUVECs, including mitochondrial shrinkage and loss of plasma membrane integrity (Figure 1D). The results of EdU incorporation assays confirmed the cytotoxic effects of both treatments, with HG and erastin significantly inhibiting DNA synthesis compared to the NG control (Figure 1C,E). Western blot analyses revealed that HG treatment downregulated GPX4 protein expression to levels comparable to those with erastin treatment while upregulating the pro‐ferroptotic enzyme ACSL4 (Figure 1F–H). These results collectively show that hyperglycemia induces endothelial cell death through ferroptosis, as indicated by ROS accumulation, mitochondrial dysfunction, proliferation inhibition, and characteristic GPX4/ACSL4 dysregulation. This mechanistic insight may position ferroptosis inhibition as a promising therapeutic strategy for diabetic wound healing.

### 3.2 ATT Reduces HG‐Induced Apoptosis in HUVECs

**FIGURE 2:**
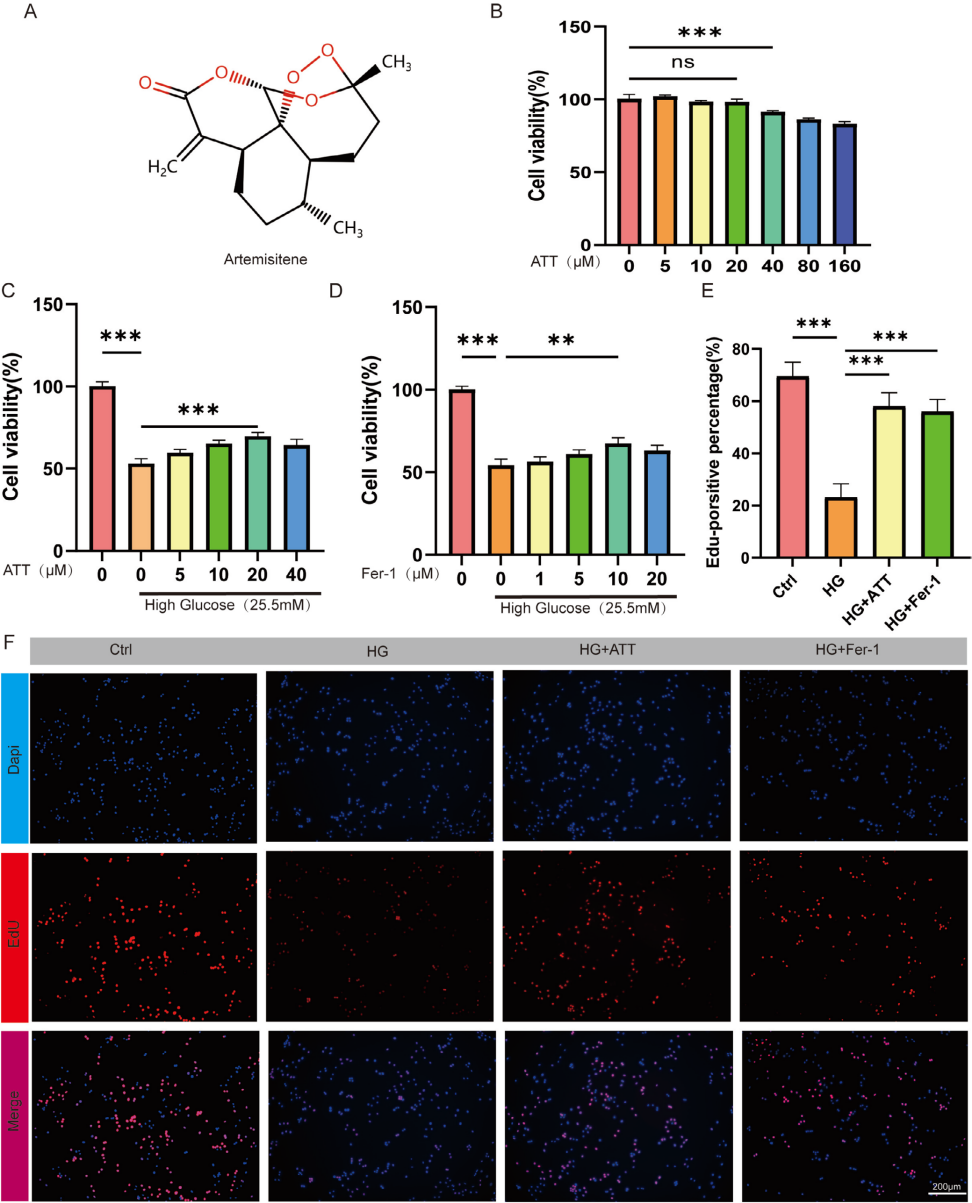
Artemisitene (ATT) has cytoprotective effects against HG‐induced damage. (A) Chemical structure of ATT. (B) Cytotoxicity assessment of ATT (0–160 μM) in HUVECs after 24 h treatment (ns, not significant; ***p < 0.001 vs. untreated; *n* = 5). (C) Dose‐dependent rescue of HG‐induced viability loss by ATT (***p < 0.001 vs. HG; *n* = 5). (D) Fer‐1 concentration (120 M) for ferroptosis inhibition. (E, F) EdU staining to evaluate DNA replication in HUVECs, highlighting EdU+ nuclei in red and total nuclei in blue. (**p < 0.01, ***p < 0.001 vs. HG; *n* = 5). Data are expressed as the mean ± SD.

ATT is non‐cytotoxic and can protect HUVECs from HG‐induced cell death. The molecular structure of ATT is shown in Figure 2A. HUVECs were treated with different concentrations of ATT for 24 h. According to our experimental results, the maximum safe concentration of ATT for HUVECs was 20 μM (Figure 2B). Under the HG condition, ATT was found to have a dose‐dependent positive effect on the survival of HUVECs (Figure 2C). To determine the optimal protective concentration of Fer‐1 against HG‐induced damage in HUVECs, different concentrations of Fer‐1 from 1 to 20 μM were tested, and a Fer‐1 concentration of 10 μM was used for subsequent rescue studies (Figure 2D). Additionally, EdU incorporation analysis showed that both ATT and Fer‐1 protected against HG‐induced damage in HUVECs and promoted HUVEC proliferation (Figure 2E,F).

### 3.3 ATT Inhibits HG‐Induced Ferroptosis in HUVECs

**FIGURE 3:**
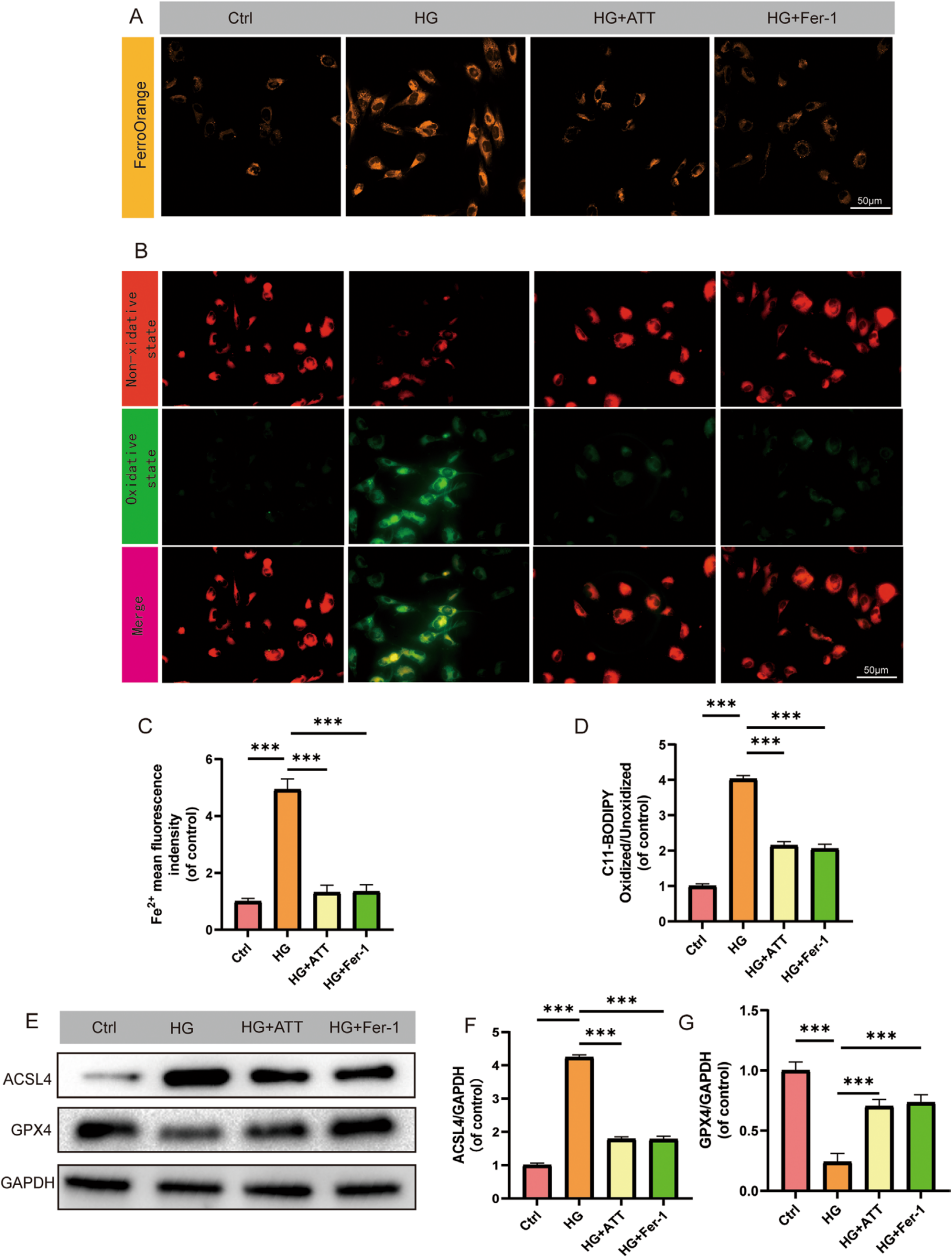
Artemisitene (ATT) inhibits HG‐induced ferroptosis in HUVECs. (A) FerroOrange fluorescence staining images of labile Fe^2+^ in the Ctrl, HG, HG + ATT, and HG + Fer‐1 groups. (B) C11‐BODIPY staining showing lipid peroxidation levels after indicated treatments. (C) Quantification of FerroOrange fluorescence signal intensity. (D) Quantitative red/green fluorescence signal ratio. (E–G) Western blot analysis of ACSL4 and GPX4 protein expression (***p < 0.001 vs. HG group; *n* = 5). Data are expressed as the mean ± SD.

Having established the biosafety profile of ATT, we investigated its ability to inhibit HG‐induced ferroptosis. Staining with FerroOrange, a specific probe for labile Fe2+, revealed elevated intracellular iron levels under HG conditions, which were effectively reversed by ATT or Fer‐1 (10 μM) (Figure 3A,C). Similarly, C11‐BODIPY staining revealed that co‐treatment with ATT or Fer‐1 markedly suppressed HG‐induced lipid peroxidation (Figure 3B,D). Western blot analysis further confirmed the ATT protective effect against ferroptosis. Additionally, HG exposure significantly upregulated the pro‐ferroptotic protein ACSL4, while downregulating the antioxidant enzyme GPX4. These pathological alterations were reversed by treatment with ATT or Fer‐1 (Figure 3E–G). Collectively, these results demonstrate that ATT effectively counteracts HG‐driven ferroptosis by regulating iron homeostasis, lipid peroxidation, and antioxidant defense pathways.

### 3.4 ATT Attenuates Mitochondrial Dysfunction in HG‐Exposed HUVECs

**FIGURE 4:**
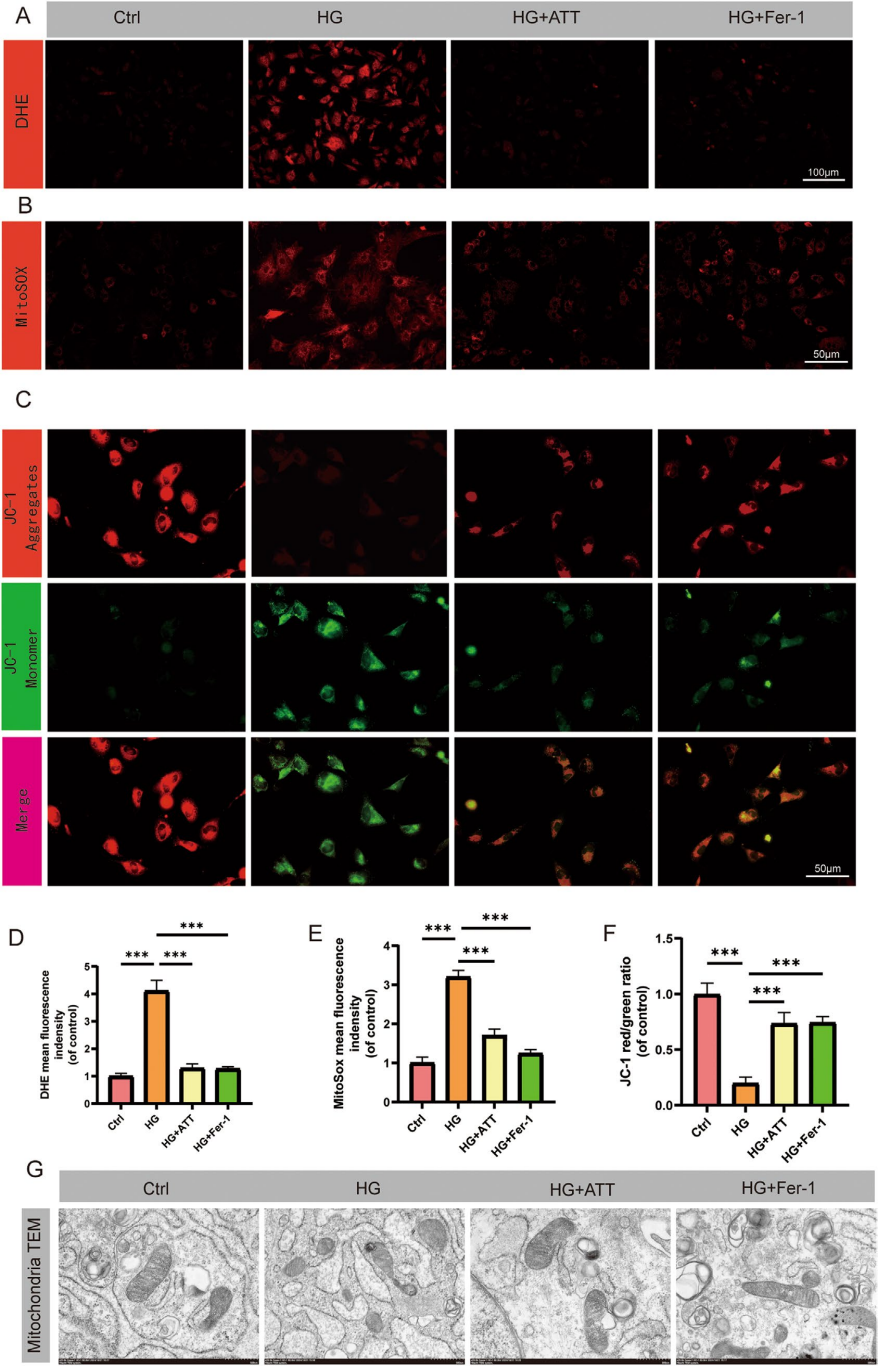
Artemisitene (ATT) improves HG‐induced mitochondrial dysfunction in HUVECs. (A) Representative DHE fluorescence staining images of intracellular ROS. (B) MitoSOX Red‐stained mtROS levels. (C) JC‐1 fluorescence (red/green) staining indicating mitochondrial membrane potential (ΔΨm). (D) Quantification of DHE fluorescence signal intensity. (E) Quantification of mtROS fluorescence signal intensity. (F) Quantitative red/green fluorescence signal ratio. (G) TEM images of mitochondrial ultrastructure. (***p < 0.001 vs. HG; *n* = 5). Data are expressed as the mean ± SD.

Our DHE images and quantitative analysis confirmed that both ATT and Fer‐1 could effectively reduce the accumulation of intracellular and mitochondrial ROS caused by HG (Figure 4A,D). We also observed that administration of ATT could restore the disrupted mitochondrial membrane potential caused by HG exposure, which was quantified by the JC‐1 fluorescent probe assay (JC‐1 assay). The green fluorescence of JC‐1 indicates a decrease in mitochondrial membrane potential and mitochondrial dysfunction, which is a signal of the early stage of cell apoptosis (Figure 4C,F). Furthermore, to assess the degree of mitochondrial‐specific oxidative damage, MitoSOX Red staining was performed. The results demonstrated a significant increase of mtROS under HG conditions, and ATT or Fer‐1 treatment reversed elevated mtROS (Figure 4B,E). Perhaps most importantly, transmission electron microscopy examination of the ultrastructure of mitochondria (Figure 4G) provided clear morphological evidence: under NG conditions, mitochondria maintained complete cristae and continuous membrane structure, while HG treatment (25.5 mM) induced characteristic ferroptosis‐related damage, including fragmentation of cristae and membrane rupture. When cells were treated with ATT (20 μM) or Fer‐1 (10 μM), this pathological transformation was significantly reversed, thereby restoring a nearly normal mitochondrial structure. In summary, our research data indicate that ATT can protect mitochondria from the decomposition of ferroptosis, thereby counteracting the toxic effects caused by the high glucose environment.

### 3.5 ATT Restores Cellular Function in HG‐Exposed HUVECs

**FIGURE 5:**
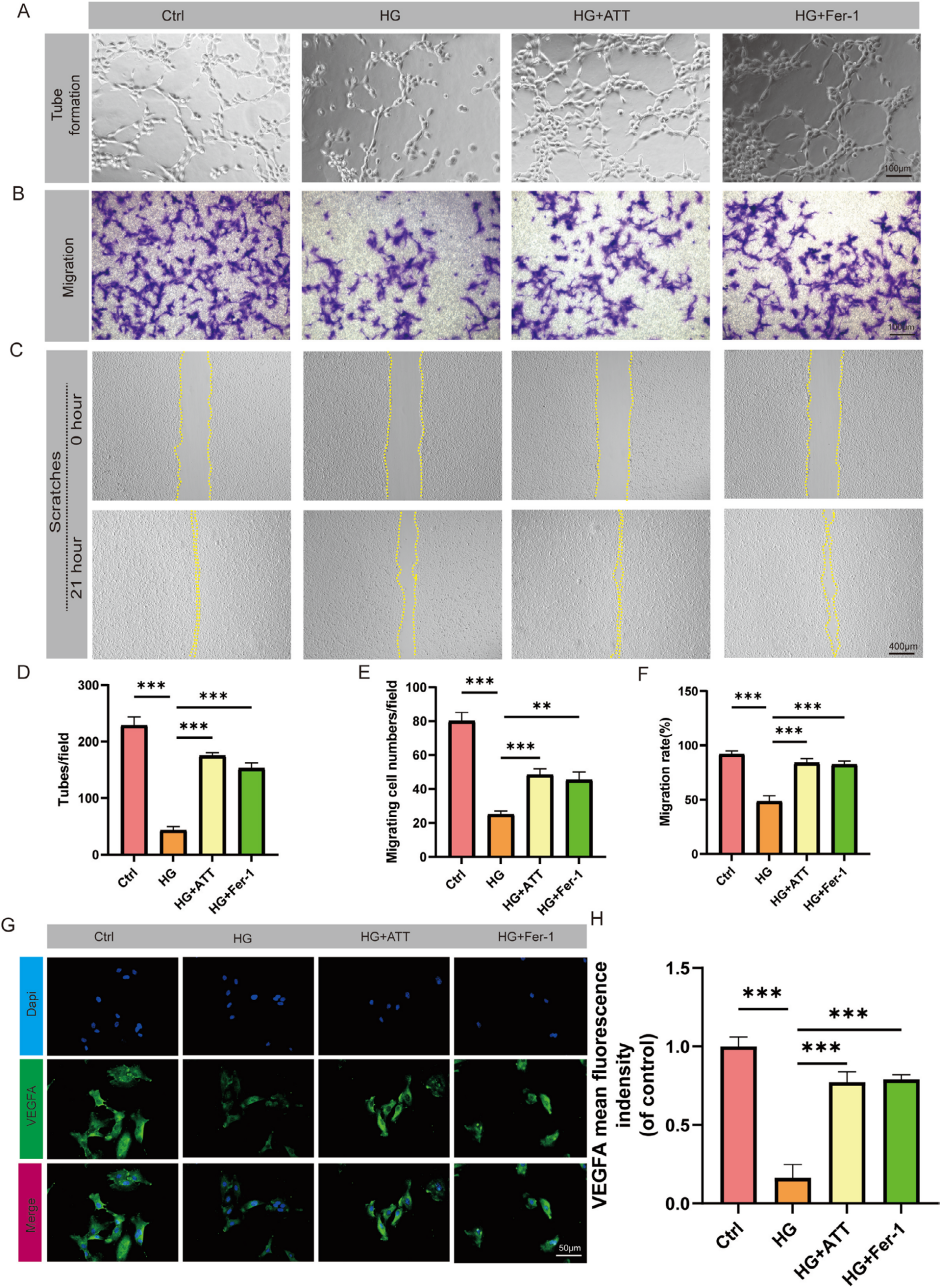
Artemisitene (ATT) restores endothelial cell function in HG‐exposed HUVECs. (A) Representative images of capillary‐like structures in tube formation assay. (B) Transwell migration assay showing hematoxylin‐stained cells. (C) Wound healing assay. (D) Quantitative analysis of tubular branches. (E) Histogram analysis of migrated cell counts. (F) Migration rates. (G) Immunofluorescence staining images of VEGFA (green) and nuclei (DAPI, blue). (H) VEGFA fluorescence signal intensity quantification (***p < 0.001 vs. HG; *n* = 5). Data are expressed as the mean ± SD.

The therapeutic effects of ATT on endothelial function were evaluated through the systematic investigation of three critical cellular processes under hyperglycemic stress. First, tube formation assays demonstrated that HG treatment severely impaired capillary‐like structure formation in HUVECs, while pretreatment with ATT or Fer‐1 effectively restored neovascularization potential (Figure 5A,D). Additionally, Transwell migration assays revealed that HG exposure significantly reduced the migratory capacity of HUVECs, as supported by the results of crystal violet staining. Treatment with either ATT or Fer‐1 reversed this inhibition, restoring cell motility to near‐physiological levels (Figure 5B,E). Furthermore, complementary wound healing assays showed that HG exposure impaired endothelial migration, which was significantly rescued by pretreatment with either ATT or Fer‐1 (Figure 5C, quantified in Figure 5F) Moreover, immunofluorescence analysis revealed that HG‐induced downregulation of vascular endothelial growth factor A (Vegfa) was effectively rescued by treatment with either ATT or Fer‐1 (Figure 5G,H), providing molecular evidence for ATT‐mediated preservation of endothelial functionality.

### 3.6 ATT Inhibits Ferroptosis in HUVECs via Nrf2/GPX4 Signaling

**FIGURE 6:**
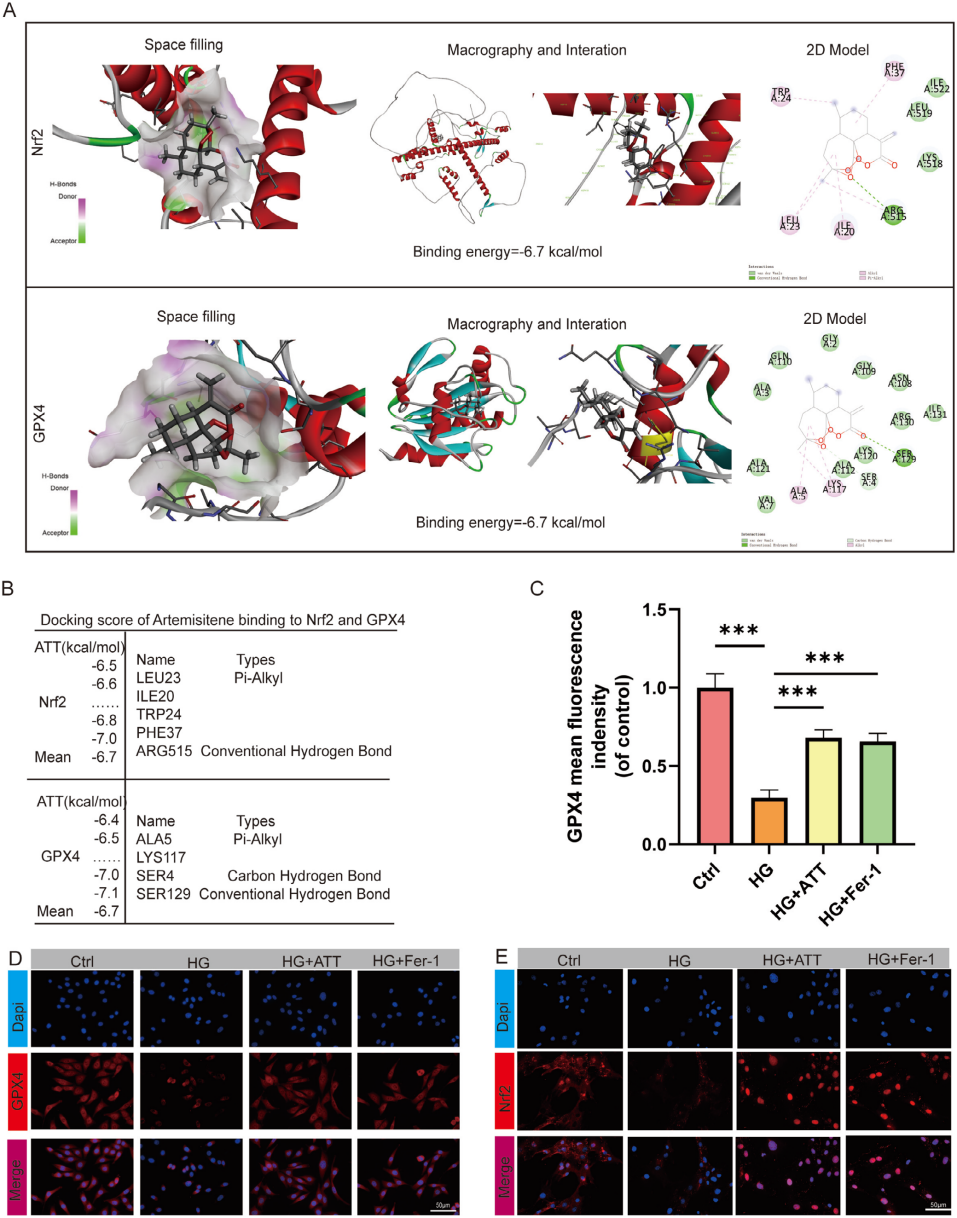
Artemisitene (ATT) inhibits ferroptosis by activating the Nrf2/GPX4 signaling pathway in vitro. (A, B) A ribbon model was used to illustrate the three‐dimensional (3D) binding model, representing protein residues. (C) GPX4 fluorescence signal intensity quantification. (D, E) Representative images of Nrf2 and GPX4 fluorescence staining in HUVECs. (***p < 0.001 vs. HG; *n* = 5). Data are expressed as the mean ± SD.

**FIGURE 7:**
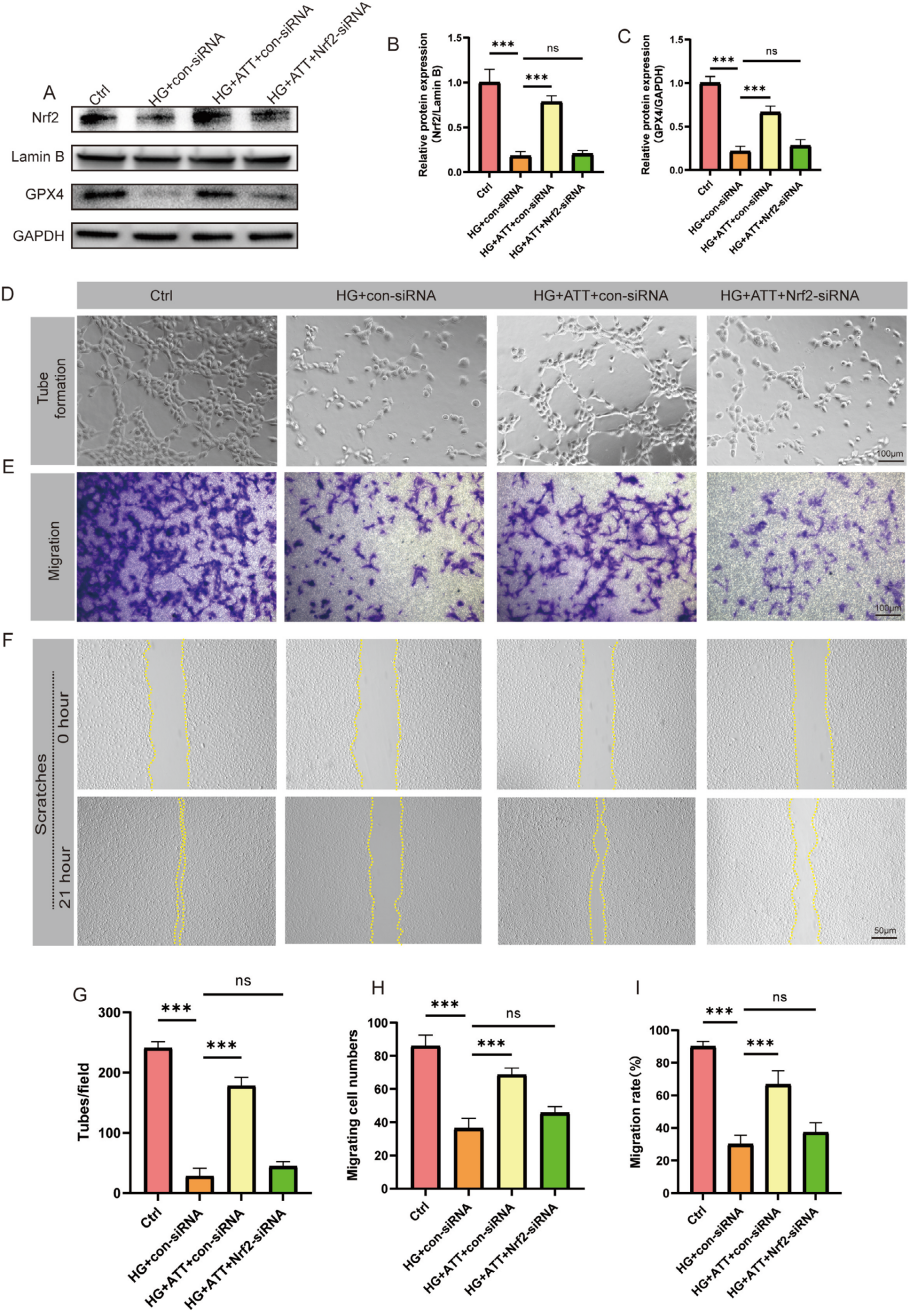
The inhibition of ferroptosis by artemisitene (ATT) and the improvement of the function of HUVECs can be regulated by *Nrf2*‐siRNA. (A–C) Western blot analysis of n‐Nrf2 and GPX4 protein expression in HUVECs. (D) Representative images of capillary‐like structures in the tube formation assay. (G) Quantitative analysis of tubular branches. (E) Transwell migration assay showing hematoxylin‐stained cells. (H) Histogram analysis of migrated cell counts. (F) Wound healing assay. (I) Migration rates. (ns, not significant; ***p < 0.001 vs. HG; *n* = 5). Data are expressed as the mean ± SD.

Molecular docking studies were performed to elucidate ATT's mechanism in diabetic wound healing. Using AI‐driven computational docking (based on structural data from PubChem and PDB, processed with AutoDock protocols), we identified high‐affinity binding between ATT and proteins on the Nrf2/GPX4 axis, with free energies of −6.7 kcal/mol (Nrf2) and −6.7 kcal/mol (GPX4). Based on molecular docking analysis, ATT forms a Conventional Hydrogen Bond with ARG515, establishes Pi‐Alkyl interactions with LEU23, TRP24, PHE37, and ILE20, and ultimately interacts with Nrf2. Subsequently, it forms Conventional Hydrogen Bond contact with SER129, a Carbon Hydrogen Bond with SER4, Pi‐Alkyl interactions with ALA5 and LYS117, and finally engages with GPX4 (Figure 6A,B). Immunofluorescence confirmed ATT rescued the Nrf2 nuclear translocation damage of HUVECs caused by HG culture, mirroring Fer‐1 effects (Figure 6D). Meanwhile, GPX4 immunofluorescence further validated ATT‐mediated restoration of GPX4 subcellular localization, and Fer‐1 treatment also increased GPX4 subcellular localization (Figure 6C,E). To determine the effect of the Nrf2/GPX4 pathway on HG‐induced ferroptosis in HUVECs, Nrf2‐siRNA was transfected into HUVECs. Western blot analyses revealed successful Nrf2‐siRNA transfection and Nrf2‐siRNA reversed ATT's effects on promoting Nrf2 and GPX4 expression (Figure 7A–C). Furthermore, Nrf2‐siRNA transfection abolished ATT's therapeutic effects, resulting in a decrease in tube formation, cell migration, and a delay oinwound scratch healing (Figure 7D–I). Together, ATT treatment may attenuate HG‐induced ferroptosis in HUVECs via the Nrf2/GPX4 pathway.

### 3.7 ATT Accelerates Diabetic Wound Healing via Redox Homeostasis

**FIGURE 8:**
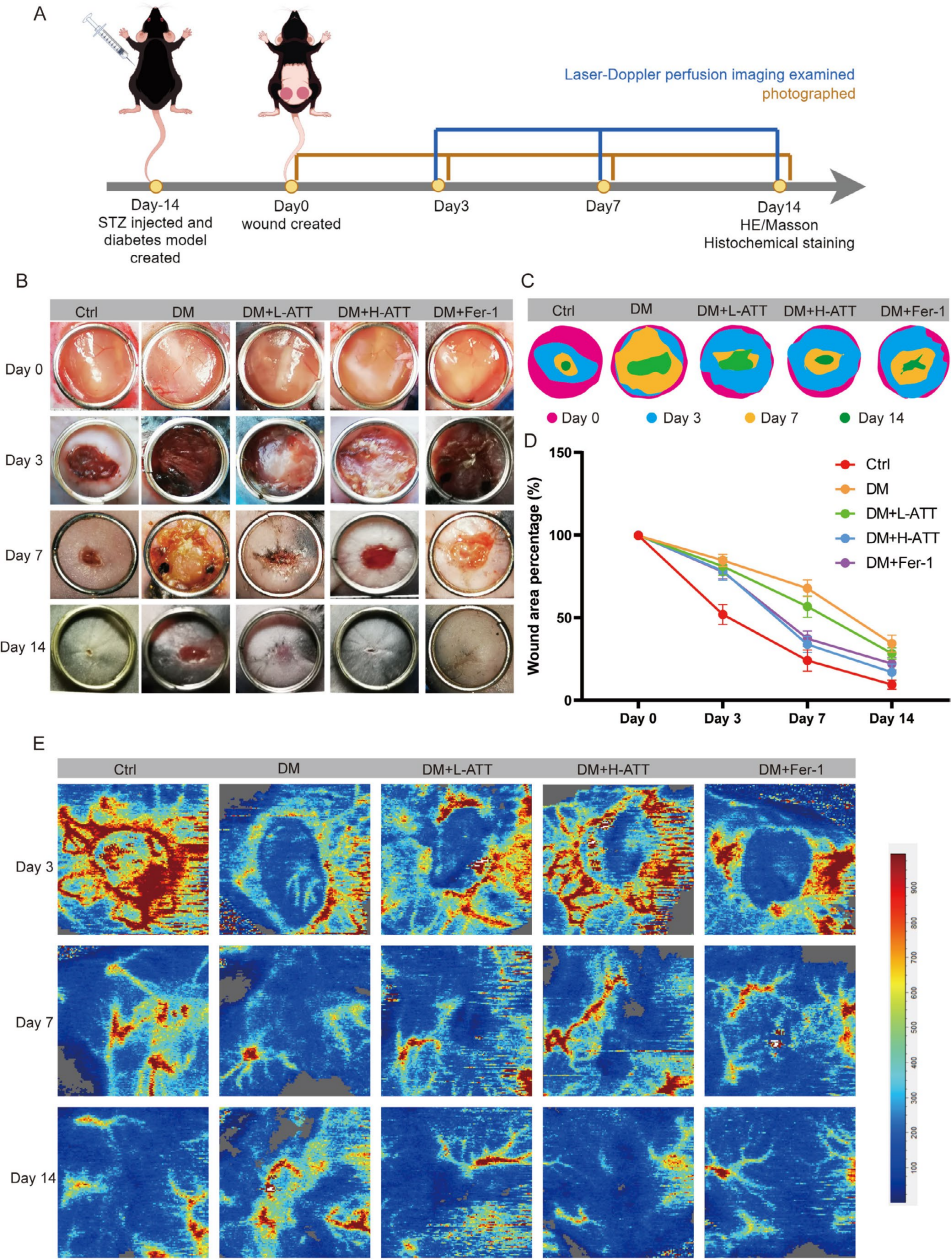
Artemisitene (ATT) restores vasculature and accelerates diabetic wound healing. (A) Surgical wound model and experimental timeline. (B) Representative wound morphology across treatment groups. (C, D) Quantified wound closure rates. (E) Laser Doppler perfusion imaging showing angiogenesis recovery (*n* = 6).

To evaluate ATT's therapeutic efficacy in vivo, full‐thickness cutaneous wounds were surgically created in streptozotocin (STZ)‐induced diabetic mice and the experimental timelines were detailed in Figure 8A. Longitudinal monitoring revealed significantly delayed wound closure in diabetic mice compared to non‐diabetic mice (Figure 8B). ATT treatment (20 mg/kg/day) markedly accelerated wound contraction, achieving healing kinetics comparable to the ferroptosis inhibitor Fer‐1 (10 mg/kg/day) at day 14 post‐injury (Figure 8B–D). Laser Doppler perfusion imaging (Figure 8E) demonstrated that DM suppressed angiogenic responses, as evidenced by diminished blood flow signals. ATT dose‐dependently attenuated this vascular dysfunction, restoring perfusion to near‐physiological levels. Fer‐1 treatment also demonstrated similar effects on blood perfusion in diabetic wounds.

### 3.8 ATT Promotes Collagen Formation and Activates GPX4 in Diabetic Wound Healing

**FIGURE 9:**
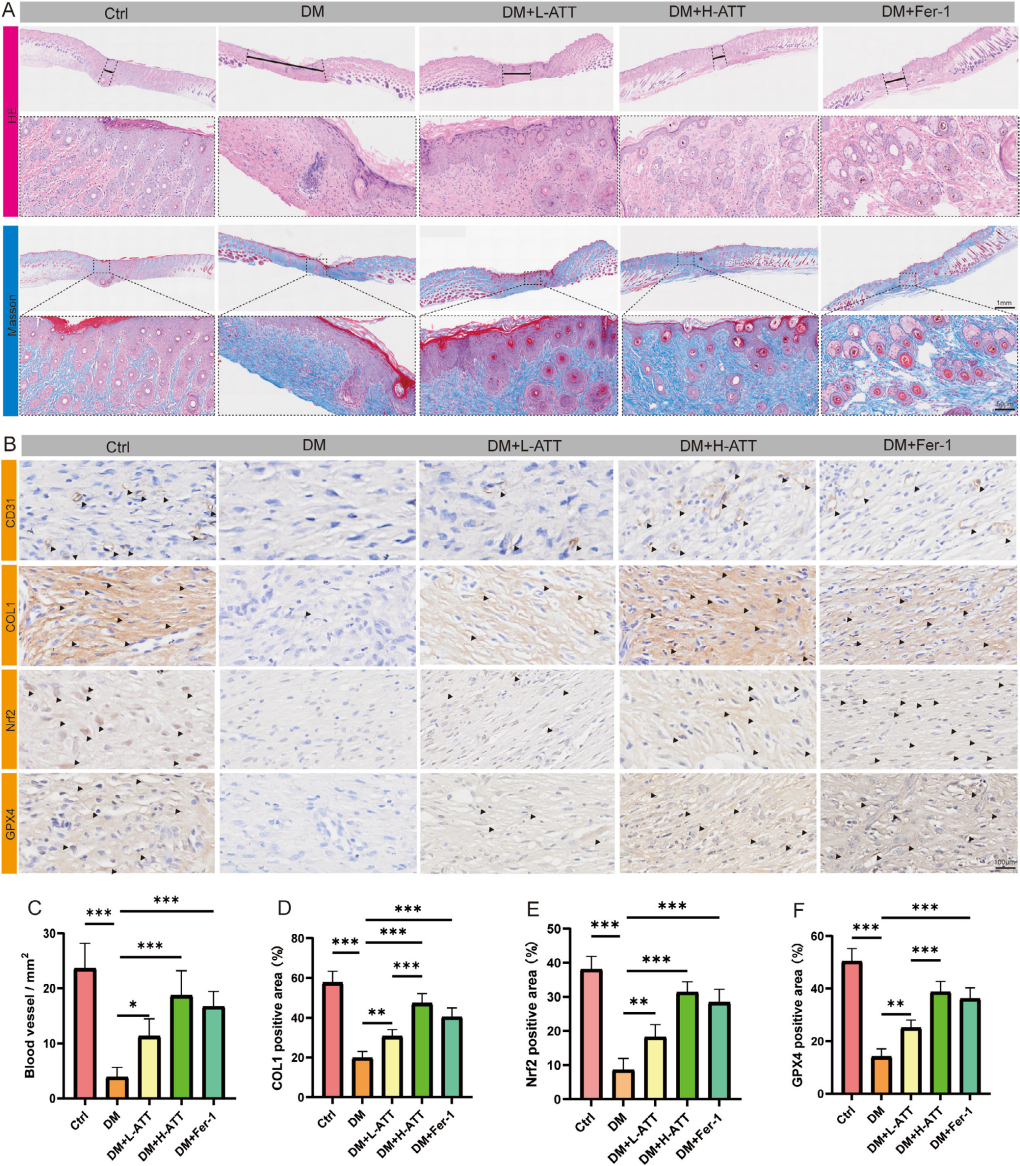
Artemisitene (ATT) enhances collagen organization and activates antioxidant pathways. (A) H&E‐stained epithelial regeneration and visualization of collagen alignment using Masson's trichrome staining. (B– F) IHC analysis of CD31 showing angiogenic networks, Nrf2 nuclear localization, COL1 and GPX4 expression. (*p < 0.05, **p < 0.01, ***p < 0.001 vs. DM; *n* = 6). Data are expressed as the mean ± SD.

To systematically evaluate ATT's therapeutic effects on tissue repair and redox regulation, we integrated multidimensional analytical approaches. Hematoxylin and eosin (H&E) staining revealed that ATT or Fer‐1 treatment significantly enhanced epithelial regeneration in diabetic wounds, forming continuous stratified epithelial structures, in sharp contrast to the fragmented epidermal layers observed in untreated diabetic wounds (Figure 9A). Masson's trichrome staining further demonstrated highly organized collagen fiber alignment in both ATT and Fer‐1 treated groups, whereas diabetic wounds exhibited disorganized and fractured extracellular matrices (Figure 9A). Immunohistochemical (IHC) analysis showed that both ATT and Fer‐1 treatments markedly increased newly formed vessels and CD31‐positive vascular density (Figure 9B,C). Collagen I (COL1) deposition analysis confirmed ATT's capacity to enhance extracellular matrix remodeling (Figure 9B,D). Mechanistically, ATT treatment promoted nuclear translocation of Nrf2 and upregulated GPX4 expression, synergistically activating antioxidant defense systems (Figure 9B,E,F). These findings demonstrate that ATT treatment may reorganize collagen and restore redox balance via the Nrf2/GPX4 axis to accelerate diabetic wound microenvironment repair.

### 3.9 ATT Enhances Angiogenesis and Activates Nrf2 in Diabetic Wound Healing

**FIGURE 10:**
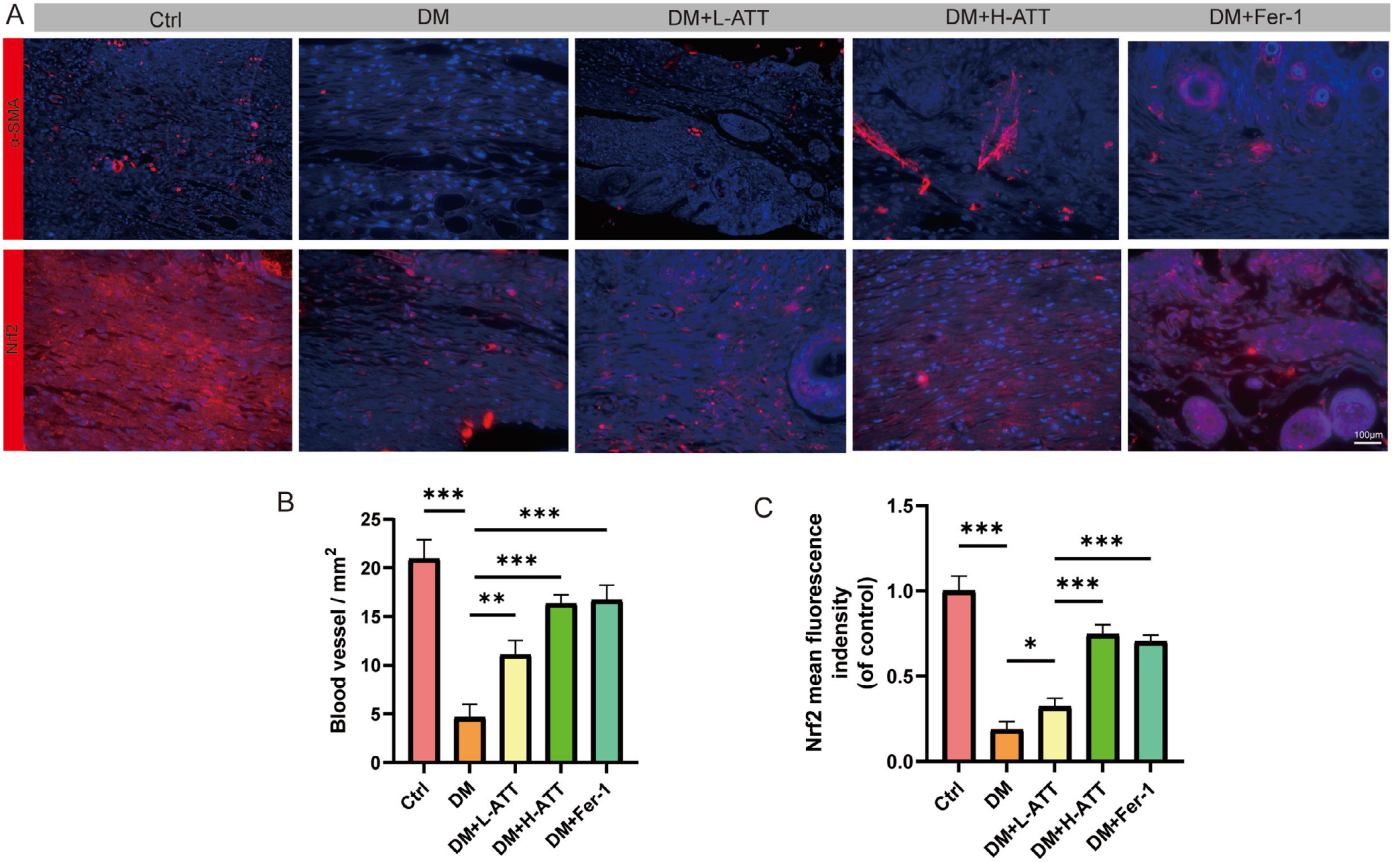
Artemisitene (ATT) promotes vascular regeneration and redox homeostasis. (A) CD31 and Nrf2 immunofluorescence staining (red) showing capillary networks. (B, C) Quantitative analysis of vascular density (*p < 0.05, **p < 0.01, ***p < 0.001 vs. DM; *n* = 6). Data are expressed as the mean ± SD.

The evaluation of the therapeutic effects of ATT on vascular regeneration by immunofluorescence analysis of α‐SMA revealed that both ATT and Fer‐1 enhanced vascular network formation in wounds, characterized by densely branched and mature capillaries at day 14 post‐injury, contrasting sharply with the sparse, fragmented vasculature observed in untreated diabetic wounds (Figure 10A,B). Mechanistically, treatment with either ATT or Fer‐1 significantly upregulated Nrf2 expression in wound tissues, with pronounced nuclear localization indicating pathway activation (Figure 10A,C). These findings indicate that ATT accelerates diabetic wound healing through improvement of angiogenesis and reduction of Nrf2‐mediated oxidative stress.

## 4 Discussion

Within the scope of this study, we used both in vitro and in vivo diabetes models to evaluate the effect that ferroptosis has on the healing process of diabetic wounds. The results of our in vitro experiments demonstrated that HG levels reduced the survival rate and migration ability of HUVECs, and increased the levels of ROS, lipid peroxidation products, and ferroptosis‐related proteins. However, treatment with either ATT or Fer‐1 effectively relieved these hyperglycemic effects. In STZ‐induced diabetic mice, treatment with ATT or Fer‐1 significantly accelerated wound closure rates, concomitant with reduced ROS/ferroptosis biomarkers and enhanced activation of the Nrf2/GPX4 antioxidant signaling pathway. These findings suggest that ATT reduces oxidative stress to inhibit ferroptosis while promoting the synthesis of pro‐angiogenic proteins, thereby improving diabetic wound healing.

In diabetic wounds, chronic hyperglycemia perpetuates inflammatory responses and excessive ROS accumulation within the wound bed, creating a self‐reinforcing cycle of oxidative stress that impedes tissue repair (Wang et al. 2024; Cano Sanchez et al. 2018). Emerging evidence indicates that HG‐induced excessive ROS production triggers mitochondrial dysfunction through maladaptive redox signaling, ultimately driving mitochondria‐dependent cell death. This underscores the therapeutic imperative to control ROS generation and preserve mitochondrial bioenergetics in DMs (Willems et al. 2015; Andrieux et al. 2021). Notably, iron dyshomeostasis has been implicated across chronic inflammatory conditions (Patil et al. 2019), with ferroptosis emerging as a key mediator of diabetic wound inflammation (Yadav et al. 2024). Recent studies have further established lipid peroxidation as a convergent mechanism linking ferroptosis to impaired healing in diabetic ulcers (Feng et al. 2022; Bi et al. 2023). The clinical relevance of ferroptosis inhibition has been corroborated by studies demonstrating improved diabetic wound healing through desferrioxamine administration (Ding et al. 2021). Characteristic mitochondrial ultrastructural changes—including cristae fragmentation and membrane disintegration—serve as pathognomonic markers of ferroptotic progression (Zheng and Conrad 2020; Liu et al. 2020), driven fundamentally by glutathione depletion and bioenergetic collapse (Li, Ming, et al. 2025). Thus, mitochondrial functional restoration represents a promising therapeutic route for ferroptosis management.

The Nrf2 transcription factor coordinates cellular antioxidant responses through transcriptional regulation of phase II detoxification enzymes (Zhang et al. 2013). GPX4, which is uniquely capable of reducing membrane phospholipid hydroperoxides, serves as the primary enzymatic barrier against ferroptosis (Jia et al. 2020). Unlike other glutathione peroxidases, GPX4 specifically neutralizes lipid‐derived ROS to prevent lipid peroxidation cascades (Yang and Stockwell 2016). GPX4 inactivation triggers lethal lipid peroxide accumulation, establishing its central role in triggering ferroptosis. Preclinical studies confirm the Nrf2/GPX4 pathway activation as an effective strategy for ferroptosis suppression in spinal cord injury and radiation‐induced neuronal damage (Wu et al. 2024; Shen et al. 2024), providing mechanistic precedent for targeting this axis in diabetic complications.

**FIGURE 11:**
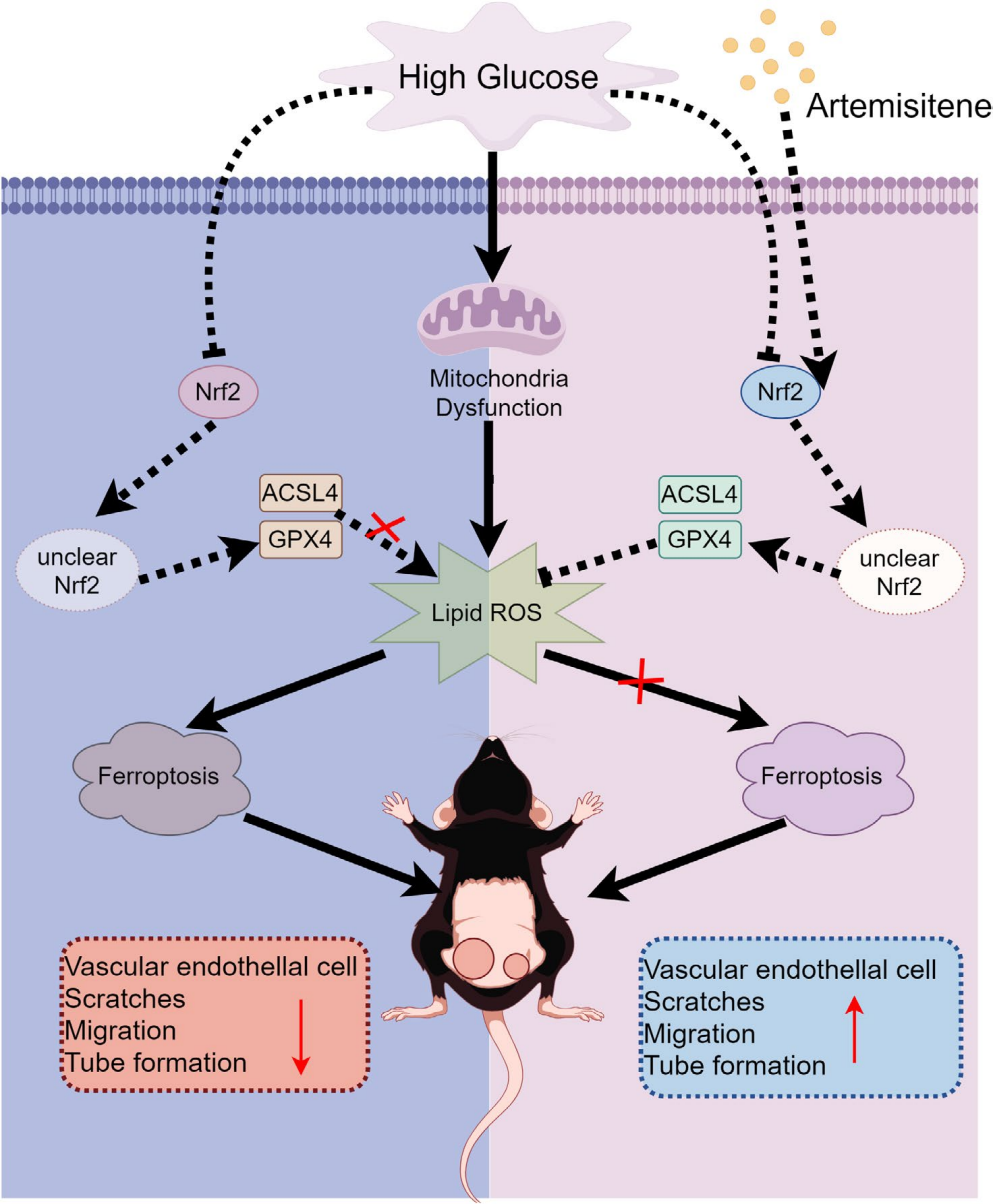
Artemisitene (ATT) accelerates diabetic wound healing by inhibiting high glucose (HG)‐induced ferroptosis in endothelial cells via Nrf2/GPX4 activation, showing dual antioxidant and anti‐inflammatory therapeutic potential.

ATT, a C‐glycosyl flavonoid derived from *A. annua* , exhibits multimodal bioactivities, including anti‐inflammatory and antioxidant properties (Hua et al. 2022). While its precise mechanisms in diabetic wound repair remain incompletely characterized, our findings position ATT as a potent Nrf2/GPX4 pathway activator that counteracts HG‐induced ferroptosis and mitochondrial dysfunction. Experimental results demonstrate that ATT promotes Nrf2 nuclear translocation, upregulates GPX4 expression, and enhances HUVEC migration/adhesion through angiogenesis‐related proteins. In vivo analyses confirm that ATT accelerates diabetic wound closure while increasing neovascularization and GPX4 expression (Figure 11). These results collectively establish ATT as a promising therapeutic candidate for diabetic wound management through coordinated ferroptosis inhibition and redox homeostasis restoration.

## 5 Conclusion

In conclusion, ATT may be a novel therapeutic candidate to accelerate diabetic wound healing by targeting the inhibition of ferroptosis. Specifically, ATT enhances mitochondrial bioenergetics and alleviates ferroptosis in HUVECs under HG conditions. Furthermore, ATT significantly enhances diabetic wound repair processes in a mice model by accelerating re‐epithelialization, augmenting angiogenesis, and restoring redox homeostasis. These beneficial effects may be related to the activation of the Nrf2/GPX4 pathway.

## Author Contributions

**Xu Honghao:** conceptualization (equal), methodology (equal), visualization (equal), writing – original draft (equal). **Bu Yitian:** project administration (equal), software (equal), validation (equal), writing – review and editing (equal). **Zhao Yuan:** data curation (equal), formal analysis (equal), supervision (equal), validation (equal). **Long Zhengyang:** conceptualization (equal), formal analysis (equal), resources (equal). **Zhou Feiya:** data curation (equal), validation (equal), visualization (equal). **Cai Leyi:** investigation (equal), methodology (equal), project administration (equal). **Gao Weiyang:** conceptualization (equal), resources (equal), software (equal). **Wang Anyuan:** resources (equal), supervision (equal), writing – review and editing (equal). **Wu Hongqiang:** funding acquisition (equal), project administration (equal), resources (equal), supervision (equal), writing – review and editing (equal).

## Conflicts of Interest

The authors declare no conflicts of interest.

## Data Availability

The data that support the findings of this study are available from the corresponding author upon reasonable request.
